# Eosinophilic Enteritis Causing Recurrent Small Bowel Obstruction: A Case Report

**DOI:** 10.7759/cureus.54355

**Published:** 2024-02-17

**Authors:** Clifford Atuiri, Wei Zhang, Christopher Cronin

**Affiliations:** 1 Internal Medicine, St. Luke's Hospital, St. Louis, USA; 2 General Surgery, St. Luke's Hospital, St. Louis, USA

**Keywords:** eosinophilic gastroenteritis, intestinal obstruction, small bowel obstruction, enteritis, eosinophilic gastrointestinal disease, eosinophilic enteritis

## Abstract

Eosinophilic enteritis is an inflammatory condition characterized by eosinophilic infiltration of the gastrointestinal tract. This case report highlights a unique presentation of eosinophilic enteritis as a cause of recurrent small bowel obstruction. The diagnosis was elusive despite extensive abdominal imaging. A histopathologic examination of a full-thickness bowel segment showing extensive eosinophilic infiltration in the muscularis propria was vital in establishing the diagnosis. This report underscores the diagnostic complexities associated with eosinophilic enteritis and the need to consider this condition as a potential cause of recurrent abdominal pain and small bowel obstruction.

## Introduction

Eosinophilic gastrointestinal diseases (EGIDs) are rare inflammatory disorders characterized by eosinophilic infiltration of the gastrointestinal tract. It can affect various parts of the gastrointestinal tract, leading to a wide range of symptoms and complications [1]. EGIDs is an umbrella term that collectively refers to a group of conditions including eosinophilic esophagitis, eosinophilic gastritis, eosinophilic enteritis, and eosinophilic colitis [2].

Data on the epidemiology of EGIDs are limited due to the rarity of the diseases. Prior survey data estimates the prevalence of EGIDs in the United States to be 26 per 100,000 persons [3]. Standardized rates and insurance claims data suggest that the diseases are much rarer: 8.4 per 100,00 and 3.3 per 100,000 for eosinophilic gastroenteritis and colitis, respectively [4].

This case report presents a unique and illustrative case of eosinophilic enteritis causing recurrent small bowel obstruction. By describing the clinical presentation, diagnostic evaluation, treatment approach, and outcome, this report aims to contribute to the existing literature on this uncommon manifestation of EGIDs. This report also underscores the importance of considering this condition in the differential diagnosis of patients with unexplained small bowel obstruction, facilitating timely intervention and optimal patient care.

## Case presentation

The patient, an adult male in his 30s, presented to the emergency room with a one-day history of right lower quadrant abdominal pain. The pain was intermittent and was associated with nausea and vomiting. The patient denied fever, chills, constipation, abdominal distention, and blood in the stool. He had no history of inflammatory bowel disease, chronic constipation, diarrhea, tumors, hernia, or abdominal surgeries. He had no allergies or recent dietary changes. 

**Figure 1:**
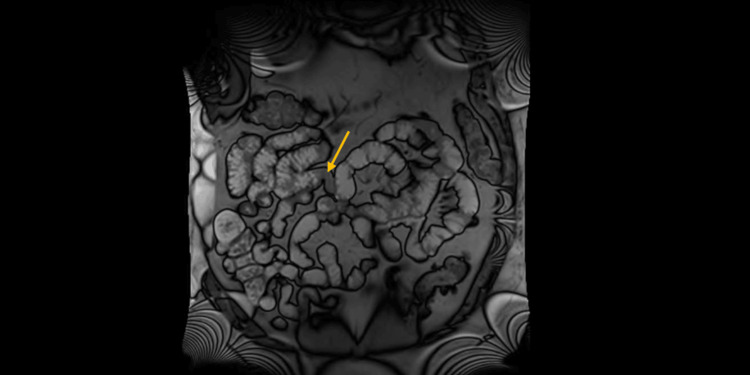
Magnetic resonance enterography showing areas of narrowing in the small bowel

The patient had experienced three other episodes of right-sided abdominal pain in the past seven years, diagnosed as small bowel obstruction with no definite etiology identified. These episodes required hospitalization, and symptoms resolved with conservative management. During those episodes, he underwent extensive evaluations with abdominal X-rays and CT scans, but no obvious cause of small bowel obstruction was identified. A magnetic resonance enterography revealed narrowing in the small bowel with hyperemia in the jejunum (Figure 1), consistent with enteritis. Between episodes, the patient was asymptomatic.

**Figure 2:**
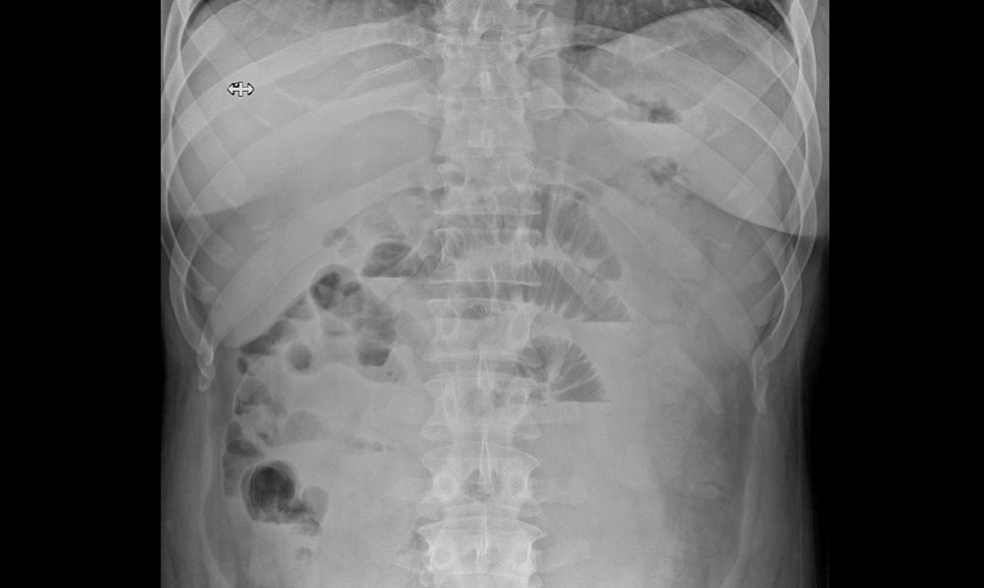
Abdominal X-ray showing multiple air-fluid levels and distended small bowel loops

**Figure 3:**
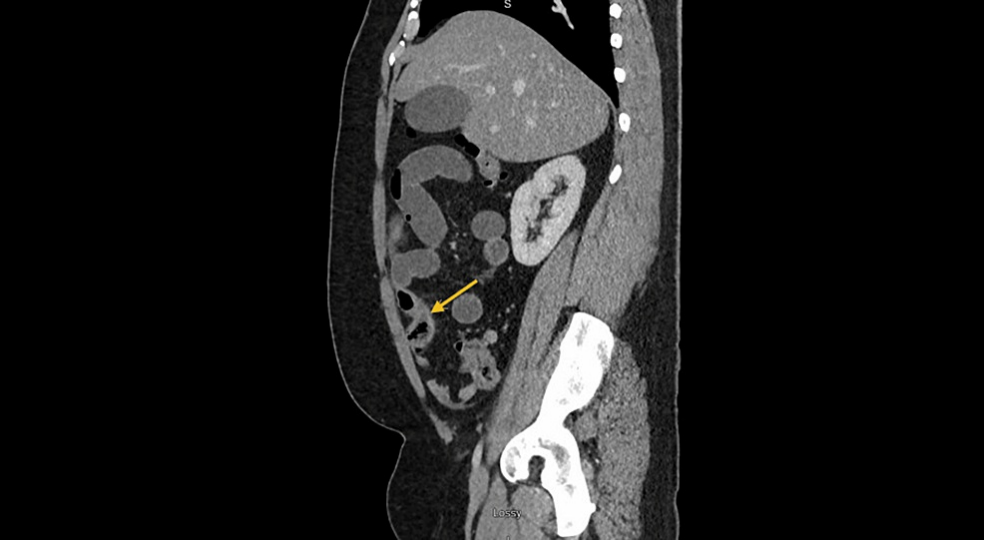
Abdominal CT scan showing dilated proximal bowel with decompression and apparent transition point (yellow arrow) in the distal small bowel

A physical examination during the acute episodes revealed abdominal distension, and tenderness in the right lower quadrant with no guarding or rebound tenderness. Laboratory investigations demonstrated an elevated WBC count (13.6 k/microL) with normal eosinophil count (0.4 k/microL) and percent (3%). Initial imaging studies, including abdominal X-rays (Figure 2) and CT scan (Figure 3), showed dilated proximal small bowel loops with decompressed distal ileum and an apparent transition point in the right lower quadrant, suggesting small bowel obstruction. There was no definite cause of the obstruction found on imaging.

The patient's symptoms persisted with conservative management. Given persistent symptoms and the patient's history of recurrent obstructions, an exploratory laparoscopy was performed, which revealed a transition point in the ileum, with dilated proximal small bowel loops and collapsed bowel loops distally. A mini-laparotomy was conducted, and bowel resection and anastomosis were carried out around the transition point.

**Figure 4:**
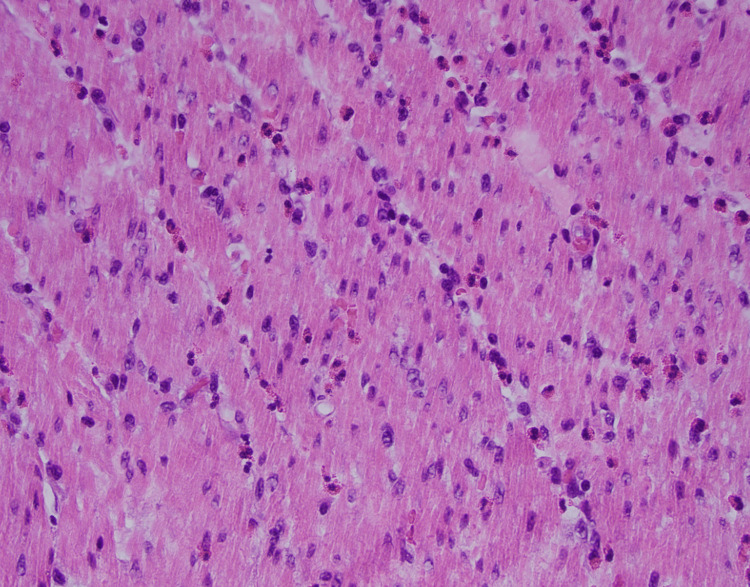
Microscopy of resected bowel segment showing abundant eosinophilia in muscularis propria (H&E x400)

**Figure 5:**
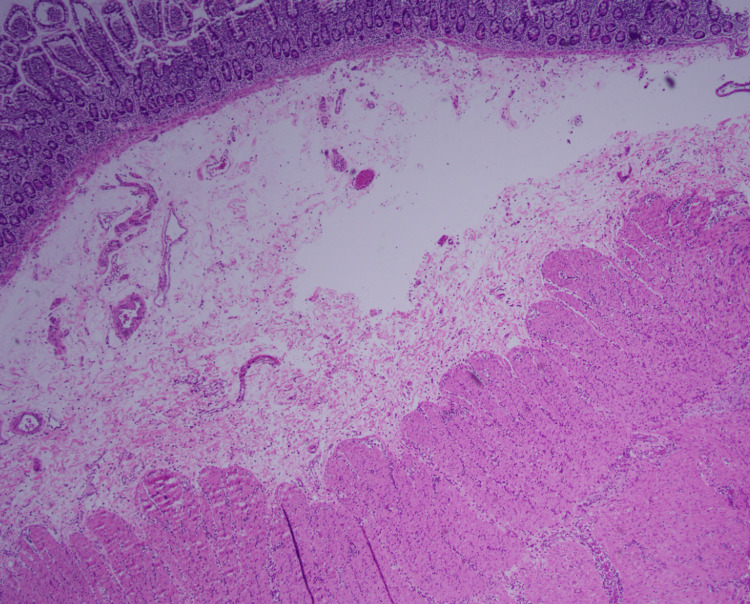
Microscopy of resected bowel segment showing mucosa, submucosa, and muscularis propria (H&E x40)

A histopathological examination of the resected bowel segment revealed an abundance of eosinophils, predominantly located in the muscularis propria (Figure 4). The mucosa appeared focally edematous with no mucosal lesions or perforations noted (Figure 5).

The patient experienced an uneventful post-operative recovery and was discharged on postoperative day five. Follow-up stool studies did not show ova or parasites.

## Discussion

This case report sheds light on the unusual presentation of an EGID as a cause of small bowel obstruction, underscoring the diagnostic challenges associated with this condition and emphasizing the significance of early recognition and appropriate management.

The patient in this case experienced recurrent episodes of intestinal obstruction, a less common yet clinically significant manifestation of eosinophilic enteritis. The cyclical pattern of symptoms, including intermittent abdominal pain and vomiting, raised suspicions of an obstructive process, prompting further investigations. Although initial imaging studies yielded inconclusive results, a histopathological examination played a pivotal role in establishing the diagnosis, revealing eosinophilic infiltration predominantly in the muscularis propria and increasing the suspicion of eosinophilic enteritis.

An EGID presents with varying clinical features depending on which layer of the bowel wall is involved. In the mucosal form, patients may exhibit vomiting, abdominal pain, diarrhea, blood in stools, malabsorption, weight loss, and protein-losing enteropathy [2,5]. The muscularis form, as observed in this case, is characterized by eosinophilic infiltration predominantly in the muscle layer, leading to bowel wall thickening and resulting in gastrointestinal obstructive symptoms. A minority of patients with eosinophilic gastroenteritis experience the serosal form, presenting with exudative ascites and higher peripheral eosinophil counts compared to the other forms [5].

The etiology of eosinophilic gastroenteritis remains enigmatic, and multiple factors have been postulated to contribute to its development [6]. Aberrant immune responses triggered by food allergens or other environmental factors have been implicated, but interestingly, about 30-50% of patients do not have any allergic associations [2]. This highlights the complex and multi-factorial nature of the condition. In this specific case, the patient did not report any known allergies or recent dietary changes, making it challenging to definitively identify the etiological factors responsible for his EGID. Notably, while eosinophilic gastrointestinal disease usually presents with elevated peripheral eosinophil counts, around 20% of patients may exhibit a normal eosinophil count [5]. The lack of specific clinical features or biomarkers makes it essential to rely on histopathological evidence of eosinophilic infiltration in the bowel wall to confirm the diagnosis. However, despite these diagnostic criteria, there may be instances where the diagnosis remains elusive due to variations in disease presentation [2].

Eosinophilic gastroenteritis is primarily a diagnosis of exclusion, relying on the evidence of eosinophilic infiltration in the bowel wall on histopathology and/or eosinophilic ascites, along with the absence of involvement in other organs and exclusion of other causes of intestinal eosinophilia [2,7].

A comprehensive differential diagnosis is crucial when managing patients with recurrent intestinal obstruction. Conditions such as Crohn's disease, gastrointestinal malignancies, and other gastrointestinal disorders may present with similar symptoms, necessitating careful evaluation and consideration of all relevant clinical information [8].

The natural course of eosinophilic gastroenteritis varies widely among patients. While some individuals experience spontaneous remission of symptoms, others may progress to severe complications, including malabsorption, malnutrition, bowel obstruction, or perforation [9]. Unfortunately, the factors influencing disease progression and outcome remain poorly understood, indicating a need for further research to establish reliable prognostic indicators and evidence-based treatment guidelines [2].

The management of eosinophilic gastroenteritis is challenging due to limited clinical trial data, and the treatment approach is largely guided by the severity of presentation and complications. For less severe cases, dietary therapy and glucocorticoid use have been suggested as potential treatment options [10]. However, for patients experiencing small bowel obstruction and/or bowel perforation, surgical intervention may be necessary.

## Conclusions

This case report adds to the limited literature on EGIDs and sheds light on the complexities of diagnosing and managing eosinophilic gastroenteritis as a cause of small bowel obstruction. The condition's diverse clinical presentation and lack of specific biomarkers demand a meticulous diagnostic approach that includes histopathological evaluation and the exclusion of other potential causes. A deeper understanding of EGIDs' etiology and natural course is essential to devise more effective management strategies and improve patient outcomes.
